# Lower Limb Injuries in Women's Handball, Protocols, and Most Common Tests: A Systematic Review

**DOI:** 10.1155/tsm2/6662321

**Published:** 2025-08-12

**Authors:** Laura Carrasco-Fernández, Manuel García-Sillero, Jerónimo García-Romero, Javier Benítez-Porres

**Affiliations:** ^1^Department of Human Physiology, Physical Education and Sport, Faculty of Medicine, University of Malaga, Málaga, Spain; ^2^Internal Medicine UGC, Victoria Virgen University Hospital, Institute of Biomedical Research of Málaga (IBIMA), Málaga, Spain; ^3^CIBER Physiopathology of Obesity and Nutrition (CIBEROBN), Carlos III Health Institute, Madrid, Spain

**Keywords:** asymmetries, handball, injuries, lower limb, power

## Abstract

The lower limbs are critical to athletic performance in handball, with strength and power being key variables. Previous studies have shown the importance of studying the rate of force development and the reactive strength index to enhance sports performance and prevent sports injuries. Given the limited research on this subject, the objective of this study was to analyze the effects of plyometric, neuromuscular, and strength training on performance and injury-related kinetic and biomechanical factors in female handball players and to identify commonly used functional and biomechanical tests for injury risk and performance assessment. This systematic review was conducted following the PRISMA guidelines and included eight clinical trials. These studies compared various training programs between the control and experimental groups, focusing on biomechanical analysis, strength training, plyometrics, neuromuscular, and standard training. The findings of this systematic review demonstrate the benefits of incorporating supplementary training into traditional handball training, specifically focusing on strength parameters and plyometric and proprioceptive exercises. Furthermore, controlling eccentric actions and addressing body asymmetries between the dominant and nondominant lower limbs reduces the risk of injuries and accelerates the rehabilitation process for injured handball players.

**Summary**

• ⁠The main kinetic and biomechanical factors are the control of body asymmetries, mainly in power and strength, and dynamic knee valgus moment between lower extremities.
• Unilateral jump tests in different planes and the isometric dynamometer were the most used in lower limb power analysis. The Y balance test was the most used test for the analysis of dynamic balance and body asymmetries.
• An additional and progressive strength, plyometric, and proprioceptive training program alongside traditional handball training is essential to meet the individual needs of each player and their requirements on the court.

## 1. Introduction

Handball is recognized as one of the Olympic sports with the highest injury rate (82.2%) [1]. This is attributed to the high physical demands of handball as a contact sport, which requires high-intensity actions such as running, jumping, pivoting, changes in speed and direction, and ball throwing [2–4].

The lower limbs play a crucial role in the performance of this high-impact sport and are the anatomical region with the highest injury prevalence in handball (58.3%), mainly affecting the knee, thigh, and ankle [5], although there is no consensus regarding a specific area within the lower limbs with the highest incidence [6,7]. Among the most frequent lower extremity injuries, traumatic injuries such as muscle strains, bruises, and sprains account for 63%, while overuse injuries, including tendinopathies, bursitis, or stress fractures, represent 37% [1]. Furthermore, previous studies have shown a 35% prevalence of recurrent injuries and contralateral lower extremity injuries in handball players [7].

In addition, adapting training programs according to the sex and gender of athletes is essential to optimize performance, prevent injuries, and promote equity in sports [8]. Female handball players have been shown to be more prone to lower limb injuries, particularly knee injuries [5]. Previous studies indicate that 6 out of 10 female handball athletes are at increased risk of sustaining knee injuries, with anterior cruciate ligament (ACL) injuries being the most common [9]. Physiological and biomechanical differences between male and female athletes may explain the disparity in prevalence and risk of lower limb injuries [10,11]. Female body morphology tends to have a greater impact on joint stability and lower limb alignment, with knee valgus movements being more common. These biomechanical patterns often hinder proper landing mechanics during jumping tasks [11]. Additionally, hormonal fluctuations related to the menstrual cycle have been identified as key factors increasing injury risk, as they affect inflammatory responses and ligamentous laxity [12,13].

As the first link in the kinetic chain, the contralateral lower extremity plays a fundamental role in maintaining balance and initiating forces transferred through the trunk during ball throwing [14,15]. However, in handball, the repeated application of unilateral loads commonly leads to the development of crossed asymmetries and body imbalances between limbs, particularly in terms of muscle strength, power output, and neuromuscular control [16]. These asymmetries mainly refer to functional differences between the dominant and nondominant limbs, which can compromise performance and increase injury risk. Previous studies have demonstrated that asymmetries greater than 7% between the dominant and nondominant limb are associated with an increased risk of sports injuries [5,17].

Additionally, other studies have observed a relationship between injury risk and rehabilitation outcomes with kinetic factors such as the rate of force development (RFD), center of pressure, time to stabilization, and reactive strength index (RSI) [5,18,19]. The RFD reflects the neuromuscular system's ability to rapidly produce in the initial phase following contraction (∆Force/∆Time) [20]. Deficits in RFD after lower limb injuries have been observed in athletes, highlighting the importance of generating high force levels in short time frames, for injury prevention and rehabilitation [20–23].

Lower extremity stiffness is also an essential factor in both sports performance and injury prevention [16]. The RSI measures leg stiffness and represents the ability to transition from an eccentric to a concentric muscular action, playing a key role in storing and reusing elastic energy during stretch-shortening cycles [20].

Previous studies have highlighted the importance of promoting eccentric actions focused on strengthening the lower limbs and enhancing stabilization, taking into account limb dominance during sports action [24]. Athletes with greater eccentric strength exhibit more effective braking strategies during sports actions and, as a consequence, better storage of muscular elastic energy [25]. Eccentric training, therefore, enhances both muscular performance and the efficient use of elastic energy in athletic movements [7,26]. These studies also highlight the effectiveness of incorporating biomechanical analysis as well as complementary neuromuscular and plyometric training alongside traditional training programs tailored for each player. This approach helps to assess and correct potential body imbalances with the aim of improving sports performance and reducing injury risk [27,28].

However, few studies have analyzed the relationship between strength and stabilization parameters of the lower extremities in women's handball. The main purpose of this review was to analyze the effects of plyometric, neuromuscular, and strength training programs on physical performance and key kinetic and biomechanical factors such as RFD, RSI, eccentric strength, and interlimb symmetry that influence injury prevention and the effectiveness of rehabilitation programs in young and professional female handball players. As a secondary objective, it aimed to identify and describe the functional and biomechanical tests used to assess injury risk and performance, highlighting their frequency of use and relevance in female handball.

## 2. Materials and Methods

### 2.1. Registration and Protocol

This systematic review was registered a priori in PROSPERO (human studies: CRD42024589379) and structured following the PRISMA guidelines [29].

### 2.2. Search Strategy and Information Sources

**Figure 1:**
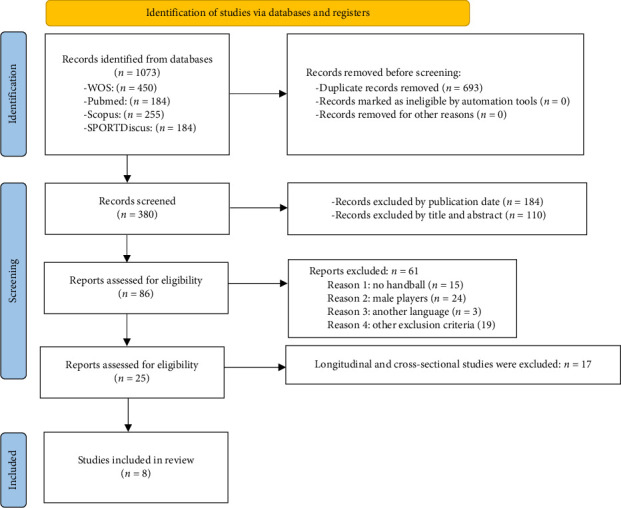
Consolidated Standards of Reporting Trials (CONSORT) flow diagram.

We conducted a systematic review of original and review studies, which were retrieved from electronic searches in the Medline (PubMed), Web of Science (WOS), Scopus, and SPORT Discuss databases.  Figure 1 provides an outline of the search methodology. The studies reviewed provide insight into the performance-related factors that should be considered in injury prevention and rehabilitation programs for lower limb injuries in handball athletes. A comprehensive analysis was conducted of all clinical trials published through 2024, and all scientific articles published in English were included with the aim of identifying the most strategies.

### 2.3. Eligibility Criteria

Inclusion criteria were defined as follows: (a) clinical trials, (b) participants were professional female handball players of any age, (c) inclusion of strength, plyometric, or neuromuscular training program, and (d) consideration of lower limb injury history.

Exclusion criteria were defined as follows: (a) scientific articles involving male handball players, (b) studies focused exclusively on upper limb training, and (c) systematic reviews.

The search algorithm combined specific terms, structured as follows: (“Rate force” OR “rate of force development” OR “RFD” OR “force” OR “strength” OR “reactive strength” OR “reactive strength index” OR “RSI”) AND (“female handball” OR “women´s handball” OR “handball”) AND (“injury” OR “injuries” OR “pain” OR “lower extremities” OR “lower extremity” OR “lower limb”).

### 2.4. Study Selection

Titles, abstracts, and full texts of the literature obtained from the databases were independently analyzed and selected by three researchers. Each researcher individually examined the titles and abstracts, taking into account the inclusion criteria for the review. Subsequently, once the initial selection was made, the full texts were thoroughly read and reviewed in light of the selected literature. Any disagreements or differences in opinion regarding the inclusion or interpretation of the articles were addressed through discussions among the three researchers, where they reached a consensus decision before finalizing the selection. This process ensured that all articles included in the review were thoroughly vetted and that the criteria for inclusion were consistently applied across all articles.

### 2.5. Equity, Diversity, and Inclusion Statement

A diverse sample of participants is included, with the aim of better understanding the different needs and realities of female handball players, regardless of their origin, socioeconomic level, or access to health services. Additionally, the researchers included a variety of perspectives in the interpretation of results in order to contribute to knowledge about women's handball, with an approach that emphasizes access to healthcare, respect for the diversity of players, and sex and gender equity. Therefore, it is essential to create an approach that contributes to improving prevention strategies and the design of more inclusive training and injury rehabilitation programs, taking into account the biological and sociocultural differences between men and women.

## 3. Results

### 3.1. Study Selection

The PRISMA diagram of the systematic search is summarized in Figure 1. A total of 1073 manuscripts were identified (450, 184, 255, and 184 from WOS, PubMed, Scopus, and Sport Discuss, respectively). Of these, 633 articles were excluded due to duplication. Following the application of the exclusion criteria, 46 studies were selected. Finally, after carrying out a comprehensive reading of the articles and taking into account the inclusion criteria; eight articles were included in the systematic review. All articles extracted from the mentioned databases were carefully evaluated based on the full text and reported supplementary data.

### 3.2. Study Characteristics

This systematic review includes eight clinical trials that examine the effectiveness of specific training programs aimed at improving sports performance and reducing the prevalence of lower limb sports injuries in elite female handball players.

In addition, Table 1 summarizes the characteristics of the eligible studies included in the systematic review. The topics presented in Table 1 are defined as follows: (a) study, (b) country, (c) number of subjects, (d) group, (e) intervention programs, (f) sport category, and (g) body composition factors. All the selected articles met the inclusion criteria and included different training programs designed to improve sports performance and reduce the risk of injuries among female handball players. Notably, the training programs of the control and experimental groups included biomechanical analysis [2], strength training, plyometric training, neuromuscular training, and standard training [34–36].

Furthermore, Table 2 presents the specific exercises that were included in the training programs carried out by the experimental groups and the control groups. This table also outlines the variables and instruments included in each program, as well as the benefits and recommendations these training regimens offer to sports and health professionals in the physical preparation protocols for elite female handball players.

#### 3.2.1. Main Outcomes

Plyometric, strength, and neuromuscular training interventions were analyzed in young female athletes, primarily within the context of women's handball. Overall, the programs showed significant improvements in physical performance, muscular balance, and injury prevention. The research included in this review mainly analyzes the following parameters and their respective tests (Table 2).

#### 3.2.2. Analysis of Anthropometric Factors and Body Composition

Weight (kg), height (cm), body mass index, muscle mass, and fat mass were measured.

#### 3.2.3. Strength and Power Analysis of the Lower Limbs

Unilateral and bilateral jump tests in different planes of movement were conducted, along with the absolute maximum torque between the hamstring (H) and quadriceps (Q) muscle groups (H/Q peak torque ratio) at the knee joint. Additionally, strength platforms, inertial sensor unit (ISU)–based biomechanical evaluations, electromyogram analysis, and isokinetic dynamometers were utilized.

#### 3.2.4. Body Stability and Balance Analysis

The Illinois and Modified Illinois change of direction tests, the star excursion balance test, and the Stork balance test were used.

#### 3.2.5. Speed Analysis

Sprint testing was performed.

Plyometric training consistently led to improvements in jump power and speed. Increases of up to 20.7% were observed in countermovement jump (CMJ) height (*p* < 0.01), along with an 18.3% improvement in squat jump (SJ) performance (*p* < 0.01). Agility also improved significantly, with a 10.4% reduction in Modified Agility T-test time (*p* < 0.001), and repeated sprint performance showed notable gains, with reductions of up to 2.76 s (*p* < 0.001). Additionally, throwing velocity increased by 18% (*p* < 0.05), and sprint times over 20 and 30 m decreased by 9.6% and 20.9%, respectively (*p* < 0.05–*p* < 0.001). Dynamic balance and upper-body strength also improved significantly following combined training interventions.

Strength training programs, including elastic band resistance and undulating strength protocols, were also effective. Sprint times over 20 m were reduced by 6.4% (*p* < 0.05), and SJ height increased by up to 7.9% (*p* < 0.05). Peak power in the CMJ improved more significantly with strength-based protocols compared to plyometric training (*p* < 0.05), while plyometric approaches led to greater gains in explosive-specific variables such as throwing velocity.

Neuromuscular training programs proved effective in injury prevention, particularly for ACL injuries. A 43% increase in vastus lateralis preactivation during cutting maneuvers was observed (95% CI: 32%–55%), suggesting improved neuromuscular control. Significant reductions were also reported in knee abduction angle (*p*=0.038), as well as in flexion and abduction moments during landing and cutting tasks (*p* < 0.05), with moderate to large effect sizes (Cohen's *d* between 0.5 and 1.4). Longer-term programs also showed increases in maximal quadriceps and hamstring strength (*p* < 0.001) and improved muscular balance, with no ACL injuries reported during the intervention periods.

Overall, the data indicate that the analyzed interventions produce significant and complementary improvements in physical performance (jumping, sprinting, and agility) and in injury prevention-related factors. Combined strategies tend to yield broader benefits by integrating explosive strength, postural control, neuromuscular activation, and functional balance components.

## 4. Discussion

This review aimed to assess the influence of various biomechanical analyses, power parameters, RFD, and RSI on the prevention of sports injuries and the design of injury rehabilitation programs, specifically for lower limb injuries in professional female handball players.

Previous studies have demonstrated the effectiveness of individualized and specific training programs for each player, taking into account the position and individual demands of each player on the court [38]. These programs include exercises focused on optimizing movement techniques used during the game, with the goal of enhancing the use and storage of elastic energy in the stretch-shortening cycles. This, in turn, promotes improvements in RFD and RSI, contributing to a reduced risk of sports injuries [21].

Based on the results of the studies analyzed in this review, the most frequent lower limb sports injuries in women's handball are associated with imbalances between the dominant and nondominant limbs [2,26], the most common being knee injuries (ACL) and ankle sprains [5,39]. Previous studies indicate the value of incorporating body composition assessments to identify asymmetries in muscle and bone mass between the dominant and nondominant limbs [40]. This approach may help prevent the development of strength and power asymmetries during jumping action and reduce the risk of sports injuries in elite handball players [41,42].

Furthermore, previous studies have highlighted the importance of integrating biomechanical and neuromuscular analysis assessments to identify risk factors for ACL injuries. Key risk factors identified for this injury include decreased maximum strength of the hip external rotators and hip flexion, increased internal rotation of the knee, and reduced preactivity of the semitendinosus muscle during the side cut [5,43,43]. These findings underscore the importance of controlling the trunk and pelvic muscles to improve hip stabilization, body balance, and lower extremity movements [40,44].

Evidence-based neuromuscular training can modify the activation strategy of the quadriceps and hamstrings during the lateral cutting movement in adolescent athletes. In this context, a decrease in preactivation of the lateral ligament of the left ventricle (VL-ST) and an increase in preactivation of the agonist of the ACL were observed, suggesting a more protective strategy for the ACL [45].

Zebis et al. and Setuain et al. observed that players with previous ACL reconstruction showed lower performance in jumping tests and mechanical efficiency, which is associated with an insufficient rehabilitation process and protective strategies during movement [2,45]. The analysis, carried out using the ISU biomechanical system and various jumping tests, revealed that players who had undergone a previous ACL reconstruction exhibited lower performance in mechanical efficiency indices and in the mediolateral force vector of the *X* axis of the lower extremity compared to their healthy counterparts [2]. This disparity in performance between limbs and between players with and without a previous ACL injury is associated with protective actions during the jumping tests performed due to an insufficient rehabilitation process [46–48].

Kim and Park did not find significant differences between high-level and low-level players in women's handball or in other sports such as basketball and soccer [1]. However, consistent with the findings of Mansouri et al., these studies highlight the importance of assessing lower limb strength and power during biomechanical evaluations of jumping in athletes with previous injuries. This is important for designing effective rehabilitation programs for women's handball players [1,5].

Furthermore, several studies have shown that elite female players often exhibit asymmetries in eccentric deceleration RFD that exceed normal ranges between the dominant and nondominant lower extremities [25,49]. Elite players are also subjected to high mechanical overloads during intense decelerations, which are associated with an increased risk of tissue damage and neuromuscular fatigue [50]. Therefore, it is essential to incorporate exercises that replicate the eccentric actions of the lower limbs, including changes of direction, sprinting, jumping and landing, braking phases, or even the demands of rapid stretch-shortening cycles that are common in elite women's handball [20].

By incorporating these exercises, it becomes possible to reduce the knee valgus angle and improve landing mechanics during both bilateral and unilateral jumping, thereby decreasing asymmetries between extremities, which is essential for the prevention of primary and secondary ACL injuries [5,46,49,51]. Nevertheless, this review does not indicate significant benefits of concentric actions in the deceleration and take-off phases of the jump compared to other sports modalities. In these sports, it has been observed that the countermovement action prior to the concentric movement improves the momentum of the concentric phase, enhancing sports performance [52,53].

Most of the studies included in this review emphasize the importance of implementing plyometric and strength training alongside traditional training programs [30,36]. Previous studies show the benefits of combining plyometric training with eccentric overload training, observing improvements in key motor skills by achieving increased muscle power, RFD, RSI, and speed in elite teams that regularly perform acceleration–deceleration actions such as jumps and direction change maneuvers requiring maximum power and strength [36,54]. The results of this review also indicate that adding just two training sessions per week is sufficient to improve mechanical power and maximal strength performance [55]. These improvements are related to nervous factors, producing an increase in neural impulses to agonist muscles, improvements in the mechanical rigidity of the muscle-tendon system, and changes in muscle architecture and muscle fiber mechanics [20,56].

Schmidt et al., like other studies in their sample, highlight the benefits of combining unilateral plyometric and proprioceptive training to prevent sports injuries in elite female players [36,57]. Similarly, Setuain et al. report that single-limb landings in women's handball involve rapid deceleration, a known risk factor for knee ACL injuries [2]. Previous research on elite basketball players also indicates a negative association between body imbalances and asymmetries with both sports performance and injury prevalence [16]. These findings underscore the importance of including unilateral exercises aimed at reducing asymmetries between the lower extremities [58].

Most of the training programs reviewed also incorporate assessments of dynamic balance, agility, and flexibility [34,59,60]. In the study by Ay et al., neuromuscular training had a positive effect on dynamic balance in female handball players within the experimental group. However, no significant improvements were found in strength, agility, flexibility, or jumping performance based on the tests used when including actions involving sudden changes of direction [34].

Other studies have compared dynamic balance levels in different directions between handball and basketball players, the latter presenting lower levels of dynamic balance in the unilateral triple hop distance jump and better levels in the horizontal vector evaluated through the unilateral triple crossover hop distance jump [34]. These findings suggest that body asymmetries in strength and balance parameters are highly individual and depend on the specific test performed during training. Notably, the Star Excursion balance test and the Y balance test (YBT) stand out as reliable dynamic tests for assessing dynamic stability in multiple planes [34,37,48].

Previous studies have demonstrated the benefits of incorporating static balance training and exercises simulating closed kinetic chain movements in handball and other sports such as soccer, athletics, or taekwondo [42,60]. Stabilization and static balance training on unstable surfaces have been shown to enhance dynamic postural control and increase isometric muscle contraction strength between the dominant and nondominant lower extremities, potentially preventing movement alterations and reducing sports injury risk [61–63].

Hammami et al. also included dynamic balance analysis in their training programs, using the YBT [59,64,65]. However, similar to other studies in handball and basketball players of younger ages, no significant improvements in dynamic and static balance were observed after a lower extremity plyometric training program [35,66]. In contrast, adult players showed improvements in stability and postural control, associated with differences in body weight among older players [67]. Furthermore, previous studies indicate that asymmetries between limbs in the anterior direction, evaluated using the YBT, are linked to decreased RSI parameters and leg stiffness, leading to decreased sports performance and increased injury risk [26].

This review presents some limitations that may be helpful in guiding future research in this area. First, there is a notable lack of studies specifically focusing on elite female handball players, which limits the availability of relevant data for developing effective performance enhancement and injury prevention strategies. Secondly, the included studies did not differentiate outcomes based on individual player characteristics, such as years of experience and court position, which restricts the specificity and practical application of the findings. Additionally, the absence of data on prior injury history further limits the interpretation of results, as previous injuries are known to influence both performance outcomes and injury risk. Future research should place greater emphasis on the development and analysis of individualized training programs, tailored to each player's specific needs, including their playing position and injury history. Furthermore, incorporating sex and gender differences into sports training programs not only tailors training to each athlete but also creates a more inclusive and respectful environment. Such an approach would contribute to more comprehensive and accurate findings, particularly in the context of injury prevention strategies and rehabilitation program design.

## 5. Conclusions

In conclusion, based on the main objective and the results of our review, the main kinetic factors that influence the prevention of lower limb injuries and improve the rehabilitation programs are the importance of controlling body imbalances and asymmetries, mainly in power and strength parameters (RFD, RSI, H:Q torque ratio, and GRF), and the dynamic knee valgus moment between the dominant and nondominant lower extremities.

This review also recommended implementing an additional and progressive training program alongside traditional handball training, which is essential to meet the individual needs of each player and their requirements on the court.

Taking into account the secondary aim of the review, among the most frequent intervention programs, strength, plyometric, and proprioceptive training stand out as the most effective. In addition, it is crucial to progressively improve the load in deceleration phases and improve eccentric actions, particularly in bilateral and unilateral landing work during jumping tasks, to improve sport performance and reduce the prevalence of injuries.

Furthermore, to improve rehabilitation and prevent lower limb injuries in elite female handball players, unilateral jump tests in different planes (CMJ, SJ, THD, and COHD) and the ISO dynamometer were the most used in RFD, RSI, and H:Q torque ratio analysis. On the other hand, YBT was the most used test for the analysis of dynamic balance and body asymmetries.

## 6. Perspectives

Compared to other sports, women's handball has less specific scientific literature on the biomechanics and physiology of female players. This systematic review investigated 8 clinical trials of elite female handball players, and the findings of this systematic review demonstrate the benefits of incorporating supplementary training into traditional handball training, specifically focusing on strength parameters and plyometric and proprioceptive exercises. Furthermore, controlling eccentric actions and addressing body asymmetries between the dominant and nondominant lower limbs reduces the risk of injuries and accelerates the rehabilitation process for injured handball players. The results reinforce the importance of including a multidisciplinary assessment between sport and health professionals, carrying out a specific and individualized analysis of each handball player with the aim of preventing sports injuries and accelerating rehabilitation programs. In addition, it is important to highlight the need for individualized assessments from the beginning of the preseason through to its conclusion. This would allow for meaningful comparisons and the analysis of potential physical changes that may serve as early indicators of an increased risk of sports injuries.

## Figures and Tables

**Table 1:** Characteristics of published studies included in the systematic review.

Author	Country	Study	Sample		Intervention program	Sport category	Age	Weight	Height
Gaamouri et al. [30]	Tunis	Clinical trial	**28**	**IG:14**	Plyometric training	Elite class	IG: 15.7 ± 0.2	63.8 ± 3.3	165 ± 0.03
				**CG:14**	Standard training		CG: 15.8 ± 0.2	624.6 ± 1.8	167 ± 1.4

Zebis et al. [31]	Denmark	Clinical trial	**40**	**IG:20**	Neuromuscular training	Elite class	IG: 15.9 ± 0.4 CG: 15.6 ± 0.5	63.8 ± 12.4 63.8 ± 9.4	168 ± 5 169 ± 6
				**CG:20**	Standard training				

Spieszny and Zubik [32]	Poland	Clinical trial	**28**	**IG:8**	Strength training	Elite class	IG: 23.1 ± 2.53	86.04	183.1 ± 4.18
				**IG:8**	Plyometric training		IG: 21.1 ± 2.17	88.69	183 ± 5.94
				**CG:12**	Standard training		CG: 23 ± 3.05	88.73	182.7 ± 5.75

Schmidt et al. [33]	Germany	Clinical trial	**19**	**IG:7**	Neuromuscular training	Elite class	IG: 15.8 ± 0.4	71.5 ± 10.2	172.7 ± 4.8
				**CG:12**	Standard training		CG: 16.0 ± 1.3	68.5 ± 10.4	174.3 ± 7.3

Ay Seher et al. [34]	Turkey	Clinical trial	**64**	**IG:32**	Neuromuscular training	Elite class	IG: 25.31 ± 5.06	66.8 ± 5.08	172.34 ± 4.77
				**CG:32**	Standard training		CG: 27.03 ± 4.97	67.8 ± 6.61	173.43 ± 4.93

Hammami et al. [35]	Tunisia	Clinical trial	**41**	**IG:20**	Plyometric training	Elite class	IG: 13.5 ± 0.3	42.6 ± 4.6	142 ± 4
				**CG:21**	Standard training		CG: 13.3 ± 0.3	42.3 ± 4.5	143 ± 4

Hammami et al. [36]	Tunisia	Clinical trial	**34**	**IG:17**	Plyometric training	Elite class	IG: 15.8 ± 0.2	64.2 ± 3.	166 ± 3
				**CG:17**	Standard training		CG: 15.8 ± 0.2	63.0 ± 3.8	167 ± 4

Hammami et al. [37]	Tunisia	Clinical trial	**36**	**IG:13**	Neuromuscular training	Elite class	IG: 15.7 ± 0.2	64 ± 3	170 ± 4
				**CG:23**	Standard training		CG: 15.8 ± 0.2	64 ± 4	167 ± 4

*Note:* The bold area represents the total sample and how it is divided into experimental and control groups in the selected studies.

Abbreviations: CG, control group; IG, intervention group.

**Table 2:** Characteristics of the training programs and recommendations proposed for injury prevention and improved sports performance.

Author	Methods		Variables	Instruments	Results and proposals
Gaamouri et al. [30]	IG: Additional plyometric training CG: Standard training	- Anthropometry - Modified agility T-half test - Vertical jumps, squat jump (SJ), and countermovement jump (CMJ) - Horizontal CMJ - Repeated sprint ability (RSA) test - RM half squat and bench press - Force velocity test	- Directional change (sprinting, shuffling, backpedalling) - Jump height - Standing long jump - Best sprint time (RSA-BT), sprint time (RSA-MT), total sprint time (RSA-TT), and fatigue index (RSA-FI) - Muscular strength - Anaerobic power, peak power, braking force, maximal velocity (Vo)	- Photocells (Microgate, Bolzano, Italy) - Infrared photocell and digital computer (Optojump System, Microgate SARL, Bolozano, Italy) - Two timing gates (Microgate Srl; Race time 2. Light Radio, Bolzano, Italy) - Standard cycle ergometer (model 894 E, Monark exercise AB, Vansbro, Sweden)	A 10-week plyometric program improved CMJ (+20.7%), SJ (+18.3%), agility (−10.4%), and sprint performance (−2.76 s RSA BT), all with statistically significant results (*p* < 0.01)

Zebis et al. [31]	IG: Additional neuromuscular training	- Side cutting manoeuvre - Neuromuscular activity - Maximal voluntary isometric strength	- Side cutting movement performed (knee joint valgus angle and maximal knee joint valgus moment) - Neuromuscular activity) in the quadriceps femoris muscle (musculus VT) and medial/lateral hamstring muscles (musculus ST and musculus BF). - Maximal voluntary isometric knee flexor strength	- 3D biomechanics 8 camera Vicon 612 system (Oxford, England), and an AMTI force plate (Massachusetts, USA) - EMG electrodes18–21 (MyoMonitor IV, Delsys, Boston, Massachusetts, USA). - Hand-held dynamometer (PowerTrack II Commander, JTECH Medical, Salt Lake City, Utah, USA)	Neuromuscular training over 12 weeks increased EMG preactivation during cutting tasks by 43% (95% IG: 32%–55%), without significant changes in knee valgus mechanics
	CG: Standard training				

Spieszny Zubik [32]	IG: Additional strength training	- Barbell jump squats, clean, and jerk dynamic bench press	- Body composition - Maximal and relative power of the lower limbs	- Force plates - Cycle-ergometer	Both strength and plyometric training improved CMJ and SJ performance (*p* < 0.05). Strength training led to greater CMJ peak power, while plyometric training increased throw velocity by 18% (*p* < 0.05)
	IG: Additional Plyometric Training	- Drop jumps, skipping, and multijumps			
	CG: Standard Training				

Schmidt et al. [33]	IG: Neuromuscular Training program (proprioceptive, plyometric, jumping, and landing exercises and core strength)	- Double-leg drop vertical landings (DLL), single-leg drop landings (SLL), and an unexpected side cut task.	- Flexion-extension, abduction-adduction and internal-external rotation of the knee. - Flexion-extension, dynamic knee valgus moment, and vertical vGRF	- Force plates 0.9 × 0.6 m (AMTI©, Watertown, MA, USA) sampled at 1000 Hz. - Light barrier (Fitlight™, VISUS GmbH, Herrenberg, Germany)	A 12-week neuromuscular training program in elite female youth handball players significantly reduced ACL injury risk factors, including knee abduction angle, knee moments, and ground reaction force (all *p* < 0.05). No changes were seen in controls
	CG: usual handball- specific training				

Ay Seher et al. [34]	IG: Neuromuscular training (strengthening, plyometric, agility, and stretching)	- Illinois agility test - Single-leg hop test - Single-leg triple jump test - Sit and reach test - Knee extensor muscle measurement - Star Excursion balance test t	- Illinois agility - Jump parameters - Flexibility - Knee extensor muscle strength - Body balance	- Hand held dynamometer recorded in Newtons (N)	In 24 weeks of the PEP program, female soccer players showed significant increases in quadriceps and hamstring strength (*p* < 0.001), improved muscle balance, 20% improved jump mechanics, and no ACL injuries occurred
	CG: regular training program				

Hammami et al. [33]	IG: Plyometric training program	- Sprint - Modified change-of-direction T test - Modified Illinois change-of direction test-Vertical jump and 5-jump test. - Back extensor strength. - Stork balance test - Y balance test	- Anthropometric - Illinois agility - Jump parameters - Dynamic balance - Power	- Optojump System; Microgate SARL - Hand dynamometer (Takei, Tokyo, Japan) - Back extensor dynamometer (Takei)	After 9 weeks of combined plyometric training, elite players showed significant improvements in sprinting, agility, jumping, strength, and balance (all *p* < 0.05)
	CG: Standard in-season regimen				

Hammami et al. [36]	IG: Plyometric training program	- Sprint - Illinois change of direction test.-Squat jump test (SJ) - Countermovement jump (CMJ), CMJ with arms (CMJA), horizontal5-jump tests (5JT), - Y balance test - Repeated sprint *T*-test (RSTT)	- Anthropometric - Illinois agility - Jump parameters - Dynamic Balance - Power	- Photocell (Microgate, Bolzano, Italy) - Infrared photoelectric (Optojump System; Bolzano, Italy) - Hand dynamometer (Takei, Tokyo, Japan) - Tanita (Tanita BF683W scales, Munich, Germany)	After 8 weeks of combined plyometric training, young female handball players improved sprint time by 8.6% (*p* < 0.01), jump height by around 11% (*p* < 0.01), and dynamic balance, demonstrating enhanced speed, power, and stability
	CG: Standard in-season regimen				

Hammami et al. [37]	IG: strength training program with elastic band training group	- Sprint - Modified Illinois change of direction test (Illinois-MT) - Vertical jump, five-jump test (5JT) - Y balance test, repeated sprint T-test (RSTT) - Strength	- Anthropometric - Illinois agility - Jump parameters - Dynamic Balance - Power	- Photocell (Microgate, Bolzano, Italy) - Infrared photoelectric (Optojump System; Bolzano, Italy) - Hand dynamometer (Takei, Tokyo, Japan) - Tanita (Tanita BF683W scales, Munich, Germany)	An 8-week elastic band strength training program improved young female handball players' 20 m sprint times by 6.4% (*p* < 0.05) and squat jump height by 7.9% (*p* < 0.05), indicating enhanced speed and power
	CG: Standard in-season regimen				

Abbreviations: CG, control group; IG, intervention group.

## Data Availability

The data used to support the findings of this study are included within the article.
